# Role of previous infection with SARS-CoV-2 in protecting against omicron reinfections and severe complications of COVID-19 compared to pre-omicron variants: a systematic review

**DOI:** 10.1186/s12879-023-08328-3

**Published:** 2023-06-26

**Authors:** Maryam Arabi, Yousef Al-Najjar, Omna Sharma, Ibtihal Kamal, Aimen Javed, Harsh S. Gohil, Pradipta Paul, Aljazi M. Al-Khalifa, Sa’ad Laws, Dalia Zakaria

**Affiliations:** Weill Cornell Medicine Qatar, Qatar Foundation, Education City, Al Luqta St. Ar-Rayyan, P.O. Box 24144, Doha, Qatar

**Keywords:** Omicron, Delta, Alpha, SARS-CoV-2, COVID-19, Effectiveness, Vaccine, Previous infection, Reinfection

## Abstract

**Background:**

The SARS-CoV-2 virus elicited a major public concern worldwide since December 2019 due to the high number of infections and deaths caused by COVID-19. The Omicron variant was detected in October 2021 which evolved from the wild-type SARS-CoV-2 and was found to possess many mutations. Omicron exhibited high transmissibility and immune evasion as well as reduced severity when compared to the earlier variants. Although vaccinated individuals were largely protected against infections in previous waves, the high prevalence of both reinfections and breakthrough infections with Omicron was observed. The aim of this review is to understand the effectiveness of previous infection on subsequent reinfection, given its significance in driving public health policy, including vaccination prioritization and lockdown requirements.

**Methods:**

A comprehensive literature search was conducted using several databases to target studies reporting data related to the effectiveness of the previous infection with SARS-CoV-2 in protecting against the Omicron variant. Screening of the studies, quality assessment and data extraction were conducted by two reviewers for each study.

**Results:**

Only 27 studies met our inclusion criteria. It was observed that previous infection was less effective in preventing reinfections with the Omicron variant compared to the Delta variant irrespective of vaccination status. Furthermore, being fully vaccinated with a booster dose provided additional protection from the Omicron variant. Additionally, most infections caused by Omicron were asymptomatic or mild and rarely resulted in hospitalizations or death in comparison to the Delta wave.

**Conclusion:**

A majority of the studies reached a consensus that although previous infection provides some degree of immunity against Omicron reinfection, it is much lower in comparison to Delta. Full vaccination with two doses was more protective against Delta than Omicron. Receiving a booster dose provided additional protection against Omicron. It is therefore clear that neither vaccination nor previous infection alone provide optimal protection; hybrid immunity has shown the best results in terms of protecting against either Omicron or Delta variants. However, additional research is needed to quantify how long immunity from vaccination versus previous infection lasts and whether individuals will benefit from variant-specific vaccinations to enhance protection from infection.

**Supplementary Information:**

The online version contains supplementary material available at 10.1186/s12879-023-08328-3.

## Introduction

The World Health Organization (WHO) was informed of a local outbreak of an atypical pneumonia with unexplained etiology in Wuhan (Hubei Province, China) in late December 2019 [1]. Quickly determined to be mediated via a novel virus (2019-nCoV) of the Coronaviridae family and the *Betacoronavirus* genus [2], the severe acute respiratory syndrome coronavirus-2 (SARS-CoV-2) elicited a major public health concern worldwide in the form of the coronavirus disease 2019 (COVID-19). Since being declared a global pandemic by the WHO on March 11, 2020 [3], the disease is estimated to be responsible for at least 630 million infections and 6.6 million deaths [4]. This tremendous degree of morbidity and mortality is mediated by viral transmission and immune escape, partly attributable to the emergence of various variants of concern (VOC) that result from rapid mutation [5]. SARS-CoV-2 VOC are defined by the United States (US) Center for Disease Control and Prevention (CDC) [6] as variants associated with “an increase in transmissibility, more severe disease (for example, increased hospitalizations or deaths), significant reduction in neutralization by antibodies generated during previous infection or vaccination, reduced effectiveness of treatments or vaccines, or diagnostic detection failures.”

Following the original Wuhan-Hu-1 strain identified in China, the D614G substitution, first prevalent in Europe, signaled one of the earliest changes in the spike (S) protein and gathered much interest as it increased infectiousness by enhancing replication and transmissibility, being present in ~ 80% of representative cases by mid-May 2020 [7–9]. Importantly, however, this substitution was not associated with resistance to neutralizing antibody (nAb) in serum samples of COVID-19 patients [10], or humoral immunity from SARS-CoV-2 mRNA vaccinees [11]. In contrast, the SARS-CoV-2 S protein N439K mutation, first sampled in March 2020 in Scotland and prevalent across more than 30 countries by January 2021, was found to have increased spike affinity for the human angiotensin-converting enzyme 2 (hACE2) receptor and evade antibody-mediated immunity; in particular, it showed resistance to several monoclonal antibodies and escape some polyclonal responses [12]. VOC Alpha (B.1.1.7), first detected in September 2020, quickly rose to become one of the prominent strains across the United Kingdom and was shown to be at least 50% more transmissible and affect a higher proportion of young adults under 20 [13]. It was also the first to correlate strongly with deletion at positions 69 and 70 of the spike protein (Δ69–70) rendering an inability to detect the S protein, dubbed S-gene target failure (SGTF). The VOC Beta (B.1.351), first detected in May 2020, signaled the dominant strain driving the second COVID-19 wave in South Africa fueled by increased transmissibility and/or immune escape [14]. VOC Gamma (P.1), first detected in Japan among travelers from Brazil in January 2021 and retraced to an earlier sample in Brazil from November 2020, was shown to be 1.7- to 2.4-fold transmissible and 21 to 46% less protected by previous infection compared to other strains; these were attributable to a triple mutation in the S protein (K417T, E484K, and N501Y) associated with increased binding to the hACE2 receptor [15, 16]. VOC Delta (B.1.617.2), first documented in October 2020 and responsible for the morbid second wave across India in early 2021, was found to be sixfold more resistant to serum nAbs from previous COVID-19 patients and eightfold resistant to vaccine-induced antibodies compared to Wuhan-Hu-1 with D614G, while displaying higher replication efficiency and increasing disease severity [17–19]. The currently dominating VOC Omicron (B.1.1.529) was first reported from Botswana and South Africa in early November 2021 and was quickly declared a VOC by the WHO, subsequently spreading to 149 countries by January 2022 [20–22].

Although vaccinated individuals were largely said to be protected against infections in previous waves, data from various sources showed the high prevalence of both reinfections and breakthrough infections with Omicron. For instance, in the initial 74 days following the first Omicron case in Iceland, 11.5% of cases were found to be reinfections, while ~ 11% of vaccinees were also found to be infected, especially in younger individuals [23]. High proportions of reinfections were similarly reported in early studies from South Africa [24], France [25, 26], Austria [27], Turkey [28], Brazil [29], and Italy [30], among others [31]. In light of this high prevalence of reinfections brought on by the Omicron VOC globally, we sought to understand the effectiveness of previous infection on subsequent reinfection by systematically reviewing all available literature on the subject, given its significance in driving public health policy, including vaccination prioritization and lockdown requirements.

## Methods

The preferred reporting items for systematic reviews and metanalysis (PRISMA) statement was used to develop the protocol of this systematic review [32].

### Information sources and search strategy

A comprehensive search was conducted to target any studies reporting the new variant of SARS-CoV-2 using the following two key words: Omicron or B.1.1.529. The following databases were searched in March 2022: PubMed, Medline, Embase, Scopus, Web of Science, Science Direct, MedRxiv, and Lens.org. All database searches utilized date limit filters of January 1, 2021 to March 6^th^, 2022 (or Current).

### Eligibility criteria

A comprehensive literature search was conducted to target medical studies that reported any data related to the effectiveness of previous infection with SARS-CoV-2/COVID-19 in protecting against the Omicron variant. No restrictions were made based on country, age, or gender. Articles without primary data, such as review articles, were excluded after removing the duplicates. Furthermore, studies that were not in English were excluded. Any studies that reported populations with positive SARS-CoV-2 infection without stratifying the data based on the variant were excluded.

### Study selection and data collection

Covidence was used for the title and abstract screening, full-text screening, and data extraction. Screening was conducted by two independent reviewers for each study and disagreements were resolved by consensus [33].

### Data items

Previous infection effectiveness (PIE) values as well as values related to the hazard ratio (HR), or rates of previously infected individuals within different cohorts (SARS-CoV-2 negative, cohorts infected with any pre-Omicron variant, cohorts infected with Omicron) were extracted in this review.

### Risk of bias and quality assessment

The quality of the included studies was assessed using the Newcastle–Ottawa Quality Assessment Scale (NOS) [34]. Quality assessment was conducted by two independent reviewers.

## Results

**Fig. 1:**
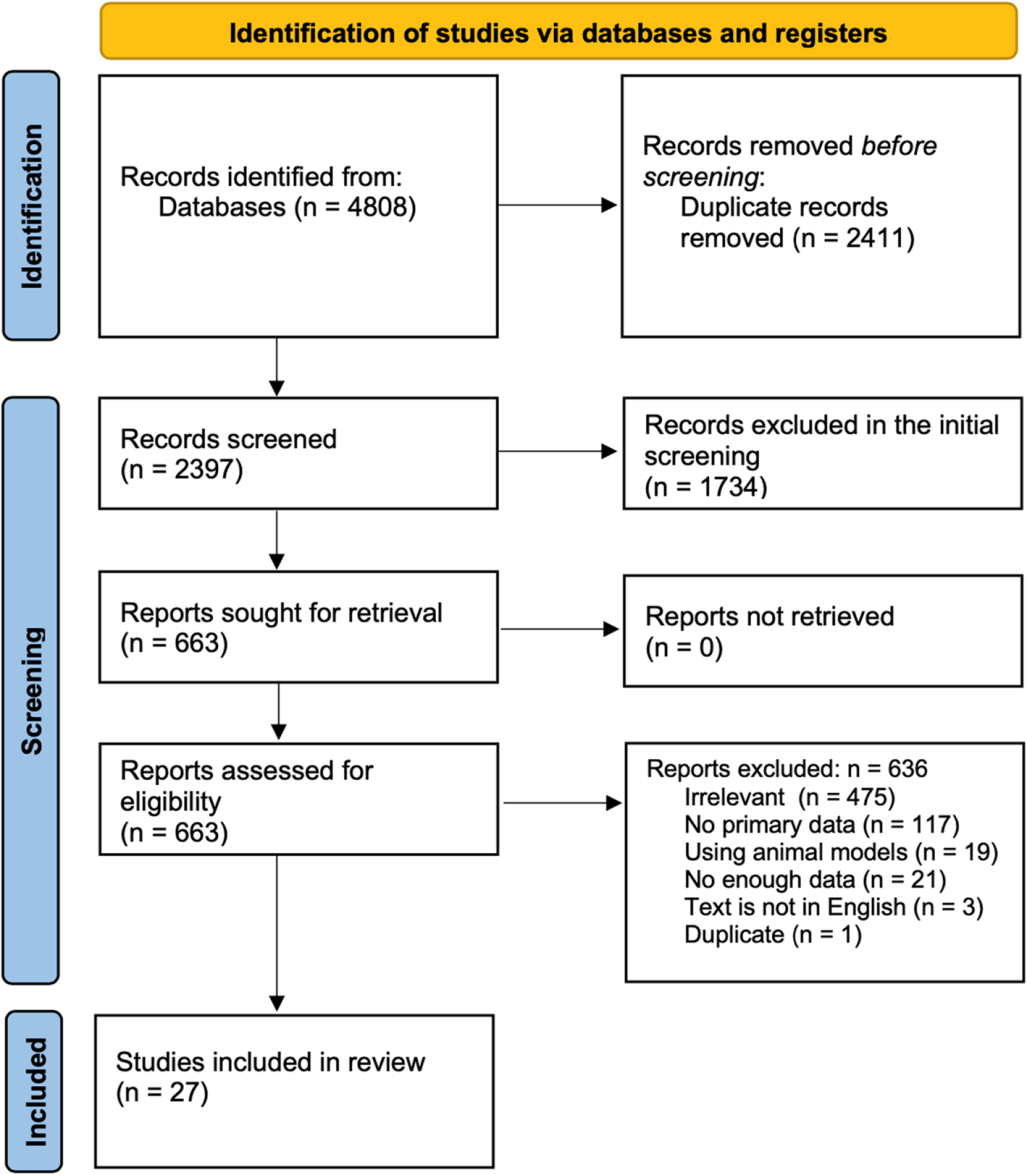
PRISMA diagram showing the study protocol

Figure 1 shows the flow diagram of the study protocol. The titles and abstracts of 2397 studies were screened after removing the duplicates, of which 663 were selected for full text screening. Only 27 studies met our inclusion criteria while 636 were excluded of which 475 were irrelevant, 21 did not have enough data, 117 had no primary data, 3 were not in English, 19 used animal models, and 1 was a duplicate of another study.

### Study characteristics and demographic data

Supplementary Table S1 summarizes the types of studies and the countries they were conducted in. Among the 27 included studies, 4 were from the US, 4 from South Africa, 1 from France, 1 from Italy, 4 from the UK, 3 from India, 2 from Denmark, 2 from the Netherlands, 2 from Qatar, 2 from Portugal, and 1 from Czech Republic. The studies included 11 cohort studies, 2 retrospective cohort studies, 2 studies used data linkages, 2 population/cohort-based surveillance studies, 2 questionnaire-based survey studies, 1 prospective cohort study, 1 test negative case control study, 1 case–control study, 1 case-case study, 1 cross-sectional study, 1 case only approach/case study, 1 registry-based study and 1 seroepidemiological study. Furthermore, supplementary Table S1 shows the NOS score for each study and summarizes all the extracted data including the PIE and the rates of previous infection (PI) in the different cohorts [35–61].

### Effectiveness of previous infection in protecting against reinfection with Omicron compared to pre-Omicron variants such as Delta

**Fig. 2:**
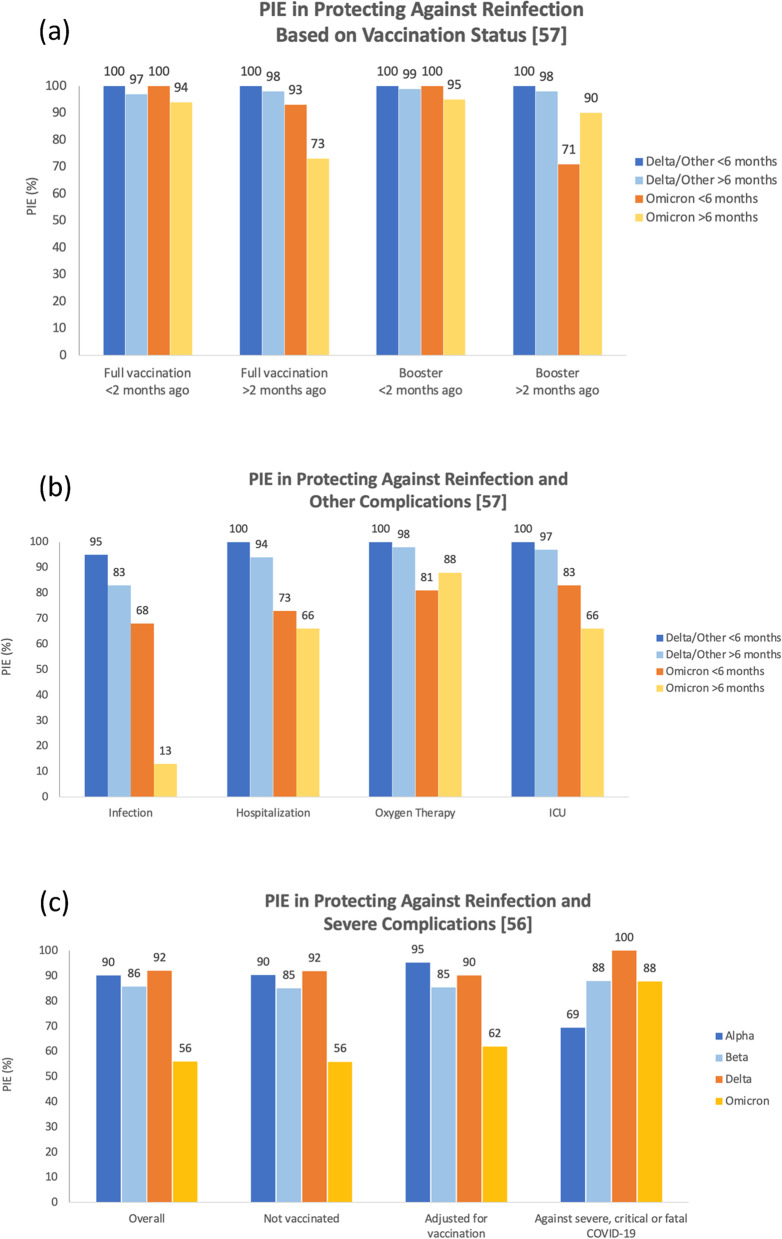
Summary of the effectiveness of previous infection (PI) with any of the SARS-CoV-2 variants in protecting against Omicron reinfections and/or its severe complications. **a** compares previous infection effectiveness (PIE) between patients fully vaccinated versus those boosted, depending on the recency of their last dosage (less than or more than two months ago) [57]. Šmíd et al. also divided subjects based on when they were previously infected (more or less than six months ago) with either Omicron or other variants, namely Delta. Data from **b** is based on the same sample used in **a**, but shows PIE against reinfection and other severe complications of COVID-19 including hospitalization, oxygen therapy, and ICU admission [57]. **c** illustrates PIE as reported by Altarawneh et al. against reinfection in the overall sample, among unvaccinated individuals, after adjusting for vaccination, and specifically against severe, critical, or fatal COVID-19 reinfection [56]

Table 1 summarizes the PIE values reported by two studies [56, 57]. Altarawneh et al. and Šmíd et al. compared the PIE in Omicron infected cohorts with cohorts infected with Alpha, Beta, or Delta variants. Both studies stratified the data based on vaccination status. The PIE was consistently lower for the protection against reinfection with Omicron compared with the Alpha, Beta, or Delta variants regardless of the vaccination status [56]. However, PI was found to be more protective against severe, critical, or fatal COVID-19 caused by the Omicron variant relative to that caused by the Alpha variant but not the Beta or Delta variants [56]. Šmíd et al., on the other hand, reported similar results where PIE was consistently lower in protecting against Omicron reinfections compared to the Delta variant at different vaccination statuses and after different time intervals [57]. However, more comparable results were reported for both variants against hospitalization less than two months after vaccination regardless of the time after the first infection (< 6 months or > 6 months) [57]. The overall PIE (regardless of the vaccination status) against hospitalization was still lower in the Omicron cohort compared to the Delta cohort. The findings of both studies are also illustrated in Fig. 2.

**Table 1:** Effectiveness of previous infection in protecting against infection and/or severe complications of COVID-19 individuals in cohorts infected with different variants of SARS-CoV-2

Study	Country/Study type	Description	Delta (or other) infected cohorts	Omicron infected cohorts
**Altarawaneh et al. **[56]	Case control study Qatar	^a^PIE overall	Alpha: 90.2% (60.2–97.6) Beta: 85.7% (75.8–91.7) Delta: 92.0% (87.9–94.7)	56.0% (50.6–60.9)
		^a^PIE adjusted for vaccine status	Alpha: 90.3% (60.4–97.6) Beta: 85.1% (74.5–91.3) Delta: 91.9% (87.8–94.7)	55.9% (50.5–60.8)
		^a^PIE vaccinated excluded	Alpha: 95.3% (66.0–99.3) Beta: 85.4% (72.4–92.2) Delta: 90.2% (81.9–94.6)	61.9% (48.2–72.0)
		^a^PIE against severe, critical, or fatal COVID-19	Alpha: 69.4% (− 143.6–96.2) Beta: 88.0% (50.7–97.1) Delta: 100% (43.3–100)	87.8% (47.5–97.1)
**Šmíd et al. **[57]	Czech Republic Database based study	^b,c^PIE against infection (overall)	Shortly after infection 95% (94–96) After 6 months 83% (82–84)	Shortly after infection 68% (68–69) After 6 months 13% (11–14)
		^b,c^PIE against infection (not vaccinated)	2–6 months after previous infection 93% (91–94) 7–10 months 91% (90%–92%) 11–14 months 86% (85–86) ≥ 14 months 79% (77–81)	2–6 months after previous infection 69% (68–69) 7–10 months 48% (46–50) 11–14 months 34% (33–35) ≥ 14 months 17% (15–18)
		^b,c^PIE against hospitalization overall	< 6 months 100% (no case) > 6 months 94% (91–96)	< 6 months 73% (55–84) > 6 months 66% (54–75)
		^b,c^PIE against hospitalization Full vaccination < 2 month ago	< 6 months 100% (no case) > 6 months 97% (91–99)	< 6 months 100% (no case) > 6 months 94% (77–95)
		^b,c^PIE against hospitalization Full vaccination > 2 month ago	< 6 months 100% (no case) > 6 months 98% (98–100)	< 6 months 93% (49–99) > 6 months 73% (78–99)
		^b,c^PIE against hospitalization Booster < 2 month ago	< 6 months 100% (no case) > 6 months 99% (99–100)	< 6 months 100% (no case) > 6 months 95% (78–99)
		^b,c^PIE against hospitalization Booster > 2 month ago	< 6 months 100% (no case) > 6 months 98% (85%–100%)	< 6 months 71% (0–96) > 6 months 90% (64%–98%)
		^b,c^PIE against oxygen therapy overall	< 6 months 100% (no case) > 6 months 98% (95%–99%)	< 6 months 81% (40%–94%) > 6 months 88% (72%–94%)
		^b,c^PIE against ICU overall	< 6 months 100% (no case) > 6 months 97% (90%–99%)	< 6 months 83% (0–98%) > 6 months 66% (15%–86%)

^a^All values are previous infection effectiveness (PIE) with 95% confidence intervals stratified by the vaccination status

^b^Effectiveness of PI (95% confidence interval) against hospitalization, oxygen therapy, or intensive care stratified by the vaccination status and time of vaccination

^c^A positive test during the first 2 months after an infection is not considered a reinfection by definition

### Rates of previously infected individuals in omicron infected cohorts compared with cohorts infected with other variants

Table 2 summarizes the rates of previously infected individuals in Omicron infected cohorts compared to cohorts infected with other variants when stratified by vaccination status. While many studies did not report the significance of the differences between the cohorts, it was observed that in three studies that reported the proportions of the naïve (not PI, not vaccinated) individuals among the Omicron and Delta cohorts, the Omicron cohort always had lower proportions of naïve patients [35, 46, 61]. Furthermore, the same three studies as well as the study by Kahn et al. reported consistently higher proportions of PI unvaccinated Omicron patients compared to the Delta patients [45]. Similar patterns were reported when PI was combined with partial or complete vaccination. However, when the rates of PI were compared in the Omicron cohorts and the cohorts with other variants of SARS-CoV-2, the Omicron cohorts had less PI individuals [35, 45, 46, 61].

**Table 2:** Rates of previously infected (PI) individuals in cohorts infected with different variants of SARS-CoV-2 stratified by vaccination status

Study	Country/Study type	Description	Negative cohorts % (n/N)	Delta (or other) infected cohorts % (n/N)	Omicron infected cohorts % (n/N) or % (n)	*P* value
**Eggink et al. **[35]	Netherlands Case only approach cohort study	Naïve (no PI no vaccine)	-	37.1% (34,765/93734)	27.4% (22,071/80615)	NR
		PI unvaccinated	-	1.4% (1295/93734)	6.5% (5253/80615)	NR
**Andeweg et al. **[61]	Netherlands Cohort study Of the infected people the presented % were:	Naïve (no PI no vaccine)	30.2% (90,945/300849)	52.8% (21,042/39889)	24.7% (3440/13915) BA.1	NR
		PI unvaccinated	4.2% (12,691/300849)	1.6% (638/39889)	5.3% (739/13915) BA.1	NR
		First start primary vaccination, then infection	1.1% (3406/300849)	0.2% (76/39889)	1.7% (240/13915) BA.1	NR
		First infection, then primary vaccination	2.3% (7002/300849)	0.3% (139/39889)	2.1% (293/13915) BA.1	NR
		PI, booster	0.3% (918/300849)	0% (2/39889)	0.5% (65/13915) BA.1	NR
**Kahn et al. **[45]	Sweden Cohort study	PI unvaccinated (0–1 dose)	-	2.7% (265/9680)	7.1% (562/7861) PI	NR
		Vaccinated (2–3 dose)	-	2.3% (94/4031)	6.3% (1376/21678) PI	NR
**Kislaya et al. **[46]	Portugal Data linkage Case-case study	Naïve (no PI no vaccine)	-	10.6% (888)	6% (315)	NR
		PI unvaccinated	-	1.3% (108)	6.2% (327)	NR
		No PI partial vaccination	-	1.3% (112)	1.3% (68)	NR
		PI partial/complete vaccination	-	0.3% (25)	0.84% (44)	NR
		No PI complete vaccination	-	88.6% (7245)	85.7% (4515)	NR
**Nunes et al. **[39]	South Africa Cohort study	PI	41.6% (101)	-	27.9% (53)	0.003
		Naïve (no PI no vaccine)	10.8% (23)	-	13.2% (23)	NR
		PI, no vaccines	6.5% (14)	-	5.2% (9)	NR
		No PI, J&J	48.6% (104)	-	56.9% (99)	NR
		PI + J&J	34.1% (73)	-	24.7% (43)	NR
**Spensley et al. **[41]	UK Cohort study	PI overall	53.5% (516/965)	-	43.4% (63/145)	0.024
		PI + ChAdOx1	-	-	46.2% (226/489)	0.75
		PI + BNT1262b2	-	-	47.2% (260/551)	0.75

N Total number of subjects in the cohort

n number of subjects who were PI or as specified in each row

Table 3 summarizes the rates of previously infected individuals in Omicron infected cohorts compared to cohorts infected with other variants without stratifying the data based on the vaccination status. All 10 studies that compared the overall rates of PI in the Omicron and Delta infected cohorts reported lower rates of PI among the Delta patients [38, 42, 49, 51–54, 58–60]. Hajjo et al. reported the clinical characteristics of the PI individuals in the Omicron infected cohort. Interestingly, 41.9% and 44.2% of the PI patients had asymptomatic or mild infections, respectively [47].

**Table 3:** Rates of previously infected (PI) individuals in cohorts infected with different variants of SARS-CoV-2 (regardless of the vaccination status)

Study	Country/Study type	Description	Negative cohorts % (n/N)	Delta (or other) infected cohorts % (n/N)	Omicron infected cohorts % (n/N) or % (n)	*P* value
**Lewnard et al. **[58]	USA Cohort study	15 December 2021 to 17 January 2022 (Documented PI)	-	0.4% (84/23305)	0.5% (1,163/222688)	NR
		3 February to 17 March 2022 (Documented PI)	-	-	0.6% (75/12756) BA.1 0.4% (7/1905) BA.2	NR
**Stegger et al. **[59]	Denmark Danish COVID-19 surveillance	Rate of second infection when PI with BA.1	-	0% (0/263)	6.46% (17/263) BA.1 17.87% (47/263) BA.2	NR
		Rate of second infection when PI with BA.2	-	0% (0/263)	0% (0/263) BA.1 1.14% (3/263) BA.2	NR
		Rate of second infection when PI with Delta	-	11.4% (30/263)	9.88% (26/263) BA.1 53.23% (140/263) BA.2	NR
		Rate of second infection when PI (overall)	-	11.4% (30/263)	12.54% (33/263) BA.1 72.24% (190/263) BA.2	NR
**Andrews et al. **[60]	UK Test-negative case–control	Rate of PI out of the Delta and Omicron	16.5% (260,073/1572621)	1.8% (3,754/204154)	11.1% (98,150/886774)	NR
**Espenhain et al. **[38]	Denmark Data from the routine Danish surveillance of COVID-19	PI	-	0.9% (160/19137)	4.3% (34/785)	NR
**Davies et al. **[42]	South Africa Cohort study	HR for PI (vs. None)	-	-	Death 1.10 (0.63—1.92) Severe hospitalization or death 0.60 (0.37—0.98) Hospitalization or death 0.28 (0.19—0.40)	-
		PI	-	Wave 3 (Delta, 20/5/2021 to 23/6/2021) 3.2% (140/4403)	Wave 4 (Omicron, 14/11/2021 to 11/12/2021) 11.3% (580/5144)	-
**Peralta-Santos et al. **[49]	Portugal Cohort Study National network group of laboratories	PI	-	1.6% (146/9397)	6.8% (449/6581)	< 0.001
**Wolter et al. **[51]	South Africa Data linkage study	^a^PI	-	4.5% (43/948) (non SGTF)	10.4% (1100/10 547) (SGTF)	0.18
**Ward et al. **[52]	UK Data linkage ^a^PI	PI	-	1% (2211/221146)	6.6% (53,724/814003)	NR
**Garg et al. **[53]	India Cohort study	PI	-	8.2% (6/182)	17.1% (14/82)	NR
**Krutikov et al. **[54]	UK Prospective cohort study	PI	-	Pre-Omicron 4.3% (17/400)	12.7% (236/1864)	< 0.0001
**Shrestha et al. **[36]	USA Retrospective cohort study	PI	-	-	2.9% (88/4585) symptomatic 1.5% (2/133) hospitalized	-
**Hajjo et al. **[47]	Jordan Questionnaire based study	Reinfected within 90 days	-	-	8.6% (43/500) of which: 41.9% (18/43) asymptomatic 44.2% (19/43) mild 2.3% (1/43) moderate 2.3% (1/43) severe 9.3% (4/43) unspecified	-
**Smith-Jeffcoat et al. **[37]	USA Cohort (convention attendees)	PI	0% (0/7)	-	6.25% (1/16)	NR
**Sharma et al. **[40]	Rajasthan, India Cohort study	PI	-	-	43.2% (126)	-
**MMWR **[43]	USA Cohort study	PI	-	-	14% (6/43)	-
**Madhi et al. **[44]	South Africa seroepidemiologic survey	PI	-	-	2.8% (195)	-
**Maisa et al. **[48]	France Questionnaire based	PI	-	-	14% (39/278) 2% hospitalized (7/294) – of these 7, 2 PI	-
**Qassim et al. **[50]	Qatar Cross sectional study	No PI	-	-	90.8% (141,839)	-
		PI < 90 days before the study	-	-	0.4% (560)	-
		PI overall	-	-	8.8% (13,803)	-

N: Total number of subjects in the cohort

n: number of subjects who were PI or as specified in each row

*SGTF* samples with S gene target failure (strongly indicative of an Omicron variant)

^a^Reinfection was defined by an individual having at least one positive SARS-CoV-2 test more than 90 days before the current positive test

Stegger et al. not only compared the rates of PI among Omicron patients with Delta patients, but they also compared the rates in the BA.1 cohorts with the BA.2 cohorts when individuals were previously infected with BA.1, BA.2, or Delta [59]. The rates were always higher for BA.2 reinfections regardless of the type of the first infection.

## Discussion

This systematic review compiled reported data that may give insight into the effectiveness of previous infection with any SARS-CoV-2 variant in protecting against Omicron infection and its severe complications compared to pre-Omicron variants. Data compiled was in the form of either the rates of previously infected individuals in cohorts infected with Omicron or other variants, or as the effectiveness of previous infection, which is expressed as the percentage of the reduction of infection, hospitalization, etc. caused by the previous infection. This is particularly important as previous infection is now considered equivalent to vaccination in some countries as part of the safety and protection measures. Furthermore, several studies stratified their data based on the vaccination status, a factor that cannot be ignored when previous infection is evaluated as a protector against infection. Therefore, below is a thorough discussion of the possible role of previous infection in protecting against the Omicron infection and its severe complications, while considering the summative effect of different levels of vaccination. We also evaluate in this review whether the high rates of reinfections during the Omicron wave might be the drive for the reduced severity of Omicron compared to the pre-Omicron variants. Furthermore, the possible mechanisms for immune evasion and the relatively higher rates of reinfections with the Omicron variant as compared to the Delta and other variants are explored below.

### Effectiveness of previous infection against Omicron as compared to Delta and the other variants

Several studies compared the rates of previously infected individuals in Omicron infected cohorts with cohorts infected with other variants without stratifying the data based on the vaccination status. Lower rates of previous infections among the Omicron patients were consistently reported compared to the other cohorts. Similarly, the studies that stratified the data based on vaccination status reported higher rates of reinfection in Omicron cohorts. For example, Eggink et al., Andeweg et al., Kislaya et al., and Kahn et al. similarly reported higher proportions of previously infected unvaccinated Omicron patients compared to Delta patients [35, 45, 46, 61]. This may indicate less protection by previous infection against the Omicron variant compared to Delta. Similar results were obtained for previously infected cohorts with partial or complete vaccination, which could be explained by the ability of the Omicron variant to escape immunity induced by either vaccination or previous infection [62, 63]. However, the Omicron cohorts had less previously infected individuals when compared with the SARS-CoV-2 negative cohorts, suggesting that prior infection may still provide a certain level of protection against Omicron infections.

While the above studies compared the rates of reinfection in cohorts with Omicron and other variants, two studies compared the effectiveness of previous infection and vaccination in protecting against infections, hospitalization, and severe outcomes caused by Omicron and the other variants. Studies conducted by Šmíd et al. and Altarawaneh et al. reported consistently lower levels of protection against reinfection with Omicron compared with the Alpha, Beta, and Delta variants, regardless of vaccination status [56, 57]. However, Altarawaneh et al. reported that previous infection provided higher protection against severe, critical, or fatal COVID-19 caused by the Omicron variant relative to that of the Alpha variant, but not the Beta or the Delta variants [56]. This study used a case–control, test-negative design to evaluate the effectiveness of previous infection in preventing new symptomatic instances of Omicron and other variants in Qatar, along with preventing death or hospitalization due to reinfection [56]. Compared to the Delta, Beta, and Alpha variants, previous infection was least effective in preventing reinfection against Omicron, with an effectiveness of 56.0% (95% CI 50.6–60.9) [56]. The effectiveness of previous infection in preventing severe COVID-19 was greatest in Delta, followed by Beta, Omicron, and Alpha. In the Omicron and Alpha variants, the greatest number of reinfected individuals progressed to severe COVID-19, and the least number occurred in those with the Beta variant, while none occurred in the Delta variant [56]. Overall, SARS-CoV-2 infection resulted in substantial protection against reinfection with the Alpha, Beta, and Delta variants, but not Omicron.

Similar results were obtained by Šmíd et. al who performed a similar analysis in the Czech population and showed that prior infection provided an overall protection of 68% against reinfection in the Omicron wave, which declined to 13% six months later, thus consistent with the waning immunity theory proposed for Omicron [57]. Furthermore, prior infection was 73% protective against Omicron-induced hospitalization, which declined after 6 months to 66% [57]. In general, it was observed that the effectiveness of previous infection was lower in protecting against Omicron infections compared to the Delta variant at different vaccination statuses and after variable time intervals.

### Previous infections and the BA.1 vs the BA.2 variants

Various subvariants of Omicron have been detected and studied, with the original being dubbed BA.1 (B.1.1.529.1), followed by the subsequent identification of some sub lineages and recombinants with distinct properties. Using compiled epidemiological data from the US, we briefly describe the relative course of the major Omicron subvariants: BA.1 reached its peak around late January 2022, accounting for > 98% of cases. BA.2 largely replaced its predecessor, and by mid-April 2022, caused ~ 75% of total cases.

Stegger et al. investigated if the Omicron subvariant BA.2 can evade immunity gained following a BA.1 infection and how this may affect the severity of COVID-19. The paper included previously infected individuals with BA.1, BA.2, or Delta and then re-infected with either of the three variants within 20 to 60 days. Although rare, BA.2 reinfections occurred shortly following a prior BA.1 infection, with 18% of the cases being BA.1-BA.2 reinfections [59]. Most BA.2 reinfections following a BA.1 or Delta infection occurred in young (< 30 years), unvaccinated individuals and resulted in mildly severe COVID-19. It was therefore elucidated that BA.2 has the inherent trait of inducing reinfections in previously infected BA.1 individuals with little or no vaccine protection [59]. However, BA.1 reinfections in those previously infected with BA.1 may simply be a residual infection due to the similarity in genomes. In those with a secondary BA.2 infection, a lower viral load was present, which may suggest a subsequent infection that is more temporary and superficial. This observation might be explained by T cell-mediated immunity acquired during the previous infection [64]. There are up to 40 non-synonymous mutations and deletions in BA.1 and BA.2, including critical changes in the spike gene's N-terminal and 74 receptor binding domains, all of which alter the immunological response [59].

Andeweg et al. conducted a test-negative study including two cohorts with previously infected individuals: Delta-Omicron BA.1 and Omicron BA.1-BA.2. Between BA.1 and BA.2, the protection afforded by vaccination and prior infection was equivalent [61]. However, previous SARS-CoV-2 infections and/or vaccination, including the booster dose, provided much less protection against Omicron BA.1compared to Delta [61]. Furthermore, protection afforded by the booster dose against Omicron BA.1 and BA.2 had dramatically declined three months following booster immunization [61]. Prior infection and primary vaccination combined offered higher protection against Omicron than either alone. Thus, individuals who had been booster vaccinated and previously infected showed the highest levels of protection. The order of vaccination and infection had little impact on the amount of protection imparted. However, the variant of the previous infection impacted the level of protection, as there was more susceptibility to Omicron despite previous infection with non-Omicron variants [61]. Previous infection provided better protection against both subvariants in the < 18 age group compared to the 19–59 age group [61]. However, individuals 60 years or older had greater protection against Omicron with the combination of vaccination and previous infection compared to younger adults. In sera from individuals who are both vaccinated and infected, broadly neutralizing antibodies against numerous variants, but not Omicron, were detected independent of the chronology of vaccination and infection [65]. Overall, both for BA.1 and BA.2, primary vaccination and pre-Omicron infections provided little protection against Omicron infection.

### Is previous infection alone enough to protect against the omicron variant?

Šmíd et. al showed that, overall, previous infection conferred greater protection against Delta than Omicron, consistently being > 95% across various combinations of vaccination and time since previous infection [57]. For the Omicron variant, when an individual was vaccinated and subsequently infected, the likelihood of being protected against a reinfection was higher than being vaccinated after a previous infection. Multiple recent studies showed that previous infection alone was not enough to protect against reinfection for either variant. Furthermore, the highest degree of protection can be acquired only if a previously infected individual is fully vaccinated against COVID-19. Altarawaneh et. al revealed similar findings wherein protection against Delta among other variants was as high as 92% both with and without accounting for vaccination status [56]. However, this protection dropped to 56% for Omicron. Shrestha et al. explored the importance of vaccination in previously infected individuals. The study was conducted over a period of one year in Cleveland Clinic, Ohio, and data was extracted based on the cumulative incidence of COVID-19 infection, symptomatic COVID-19, and hospitalization [36]. It was concluded that both vaccination and previous infection provide protection against COVID-19 infection in comparison to those with no previous infection and vaccination [36]. Likewise, Šmíd et al. suggested that a combination of both previous infections offering immunity as well as vaccination worked best together in delivering the greatest level of protection. However, either factor alone did not offer substantial protection [57]. This study further investigated the usage of any kind of oxygen therapy and the need for ICU admission in the data set. The paper found evidence that the Omicron variant is less responsive to immunity produced by vaccination and any previous infections, specifically when compared to Delta which proves to be more severe than Omicron but more responsive to vaccination and previous infections [57].

Despite initial concerns of various adverse events stemming from accelerated production and distribution of the various SARS-CoV-2 vaccines [66–69], they have undoubtedly been the most effective shield against this pandemic [70–72], estimated to have prevented 20 million deaths in the first year of roll-out alone [73]. Vaccines were shown to act as a booster in those previously infected by eliciting a similar titer count compared to infection-naïve individuals receiving 2 doses [74, 75], while also providing more durable protection in such individuals [76, 77]. Thus, although mRNA vaccines have been shown to elicit increased adverse events in patients with a history of COVID-19 infection [78, 79], boosters have been recommended due to their superior role in preventing hospitalization (from Delta) compared to non-recent full vaccination, prior infection, or vaccine-enhanced prior infection [80].

### Duration of immunity caused by previous infection

A study by Flacco et. al looked at an Italian population to analyze the length of immunity against Omicron proceeding a primary infection. The duration that immunity caused by previous infection may protect individuals from reinfections was demonstrated to be relatively long, over 12 months, as the reinfection rates were similar following primary infection and after 18–22 months from primary infection [55]. Madhi et al. suggested that natural infection may induce a diverse polyepitopic cell-mediated immune response that targets the spike protein, nucleocapsid protein, and membrane protein. As a result, cell-mediated immunity is likely to be more durable than neutralizing antibody-mediated immunity [44]. Furthermore, natural infection induces robust memory T-cell responses, including long-lived cytotoxic (CD8 +) T cells [81–83].

Kurahashi et al., reported that 2 doses of mRNA vaccination induced cross-neutralizing activity against omicron in SARS-CoV-2 convalescent patients [84]. Furthermore, Andeweg et. al evaluated patients in the Netherlands to determine whether primary vaccination, booster doses, or previous infection protected against Omicron [61]. It was seen that waning immunity was very apparent with full primary vaccination, resulting in a drop of protection from 70% to nearly 32% in 30 weeks for Omicron compared to 78–95% to 71–84% for Delta [61]. This protection was slightly enhanced with a booster dose, reaching 69% effectiveness against Omicron in the first month and 51% in the fourth month [61]. Interestingly, the study showed that this decrease was not seen in individuals who both received a booster dose and were previously infected [61]. This is critical as it suggests that while there is a concept of waning immunity over time in Omicron [85], can be prevented by being boosted against COVID-19, particularly if the individual was previously infected.

### Reinfections: from scarcity to commonplace

Prior to the rise of Omicron, reinfections following a previous infection (most likely due to an earlier variant) were scarce, with such cases being carefully studied to identify potential predisposing risk factors [86]. At the time, reinfections were found to be less severe than primary infections, likely mediated by protection conferred by immunity caused by previous infection against reinfections with Alpha and Beta VOC [62, 87]. However, the rise of Omicron and its subvariants saw robust immune evasion from not only convalescent plasma obtained from prior COVID-19 patients, but also from vaccinees, including those boosted, and even against monoclonal antibodies [88–91].

The rapid infectiousness of Omicron and its subvariants is mediated by more than thirty amino acid mutations in the S protein, of whom fifteen are in the receptor binding domain (RBD), and at least three further mutations of the furin cleavage site [92, 93]. N-terminal protein (NTD) mutations of the S protein have shown to be responsible for significant evasion from NTD-targeting nAbs [94], whereas some RBD mutations have been associated with immune evasion, being primarily responsible for vaccine breakthrough infections and re-infections; nevertheless [95]. Despite these hypermutations of the S protein, Omicron has been shown to retain, and in some cases, increase its affinity for hACE2 receptor [96]. Mutations at the S1/S2 protein border have been linked to increased fusogenicity, which in turn has been linked to pathogenicity. Delta was revealed to possess both increased fusogenicity and pathogenicity, whereas Omicron BA.1 exhibited both lower fusogenicity and milder pathogenicity [97]. The mutations in the furin cleavage site also mediated important roles in infectivity and evasion of immunity [98]. Compared to previous variants, Omicron reportedly also contained lower viral copy numbers in lung epithelial cells but increased viral copy number in the nasal airway epithelial cells. This, coupled with a switch in Omicron’s infection mechanism, from syncytia formation to endosomal fusion and thus signaling an increase in the number of potentially affected cell types, further provided evidence of its increased transmissibility but decreased severity [99]. This was further supported by statistically significant reductions in hospital and ICU admissions, need for oxygen therapy, and death in Omicron-infected patients compared to those infected with other lineages as reported by several studies [100].

In addition to the reduced virulence of the Omicron variant, the role of prior infection in minimizing the severity of Omicron reinfections was investigated in several studies. Given data suggesting that the Omicron variant has high immune escape capabilities compared to other variants [24], Wolter et al. proposed that many of the Omicron infections were likely to be reinfections rather than first-time infections [51]. With reinfections being less severe, this could in part entail the observed reduced severity in the Omicron cohort [63].

## Conclusion and recommendations

**Fig. 3:**
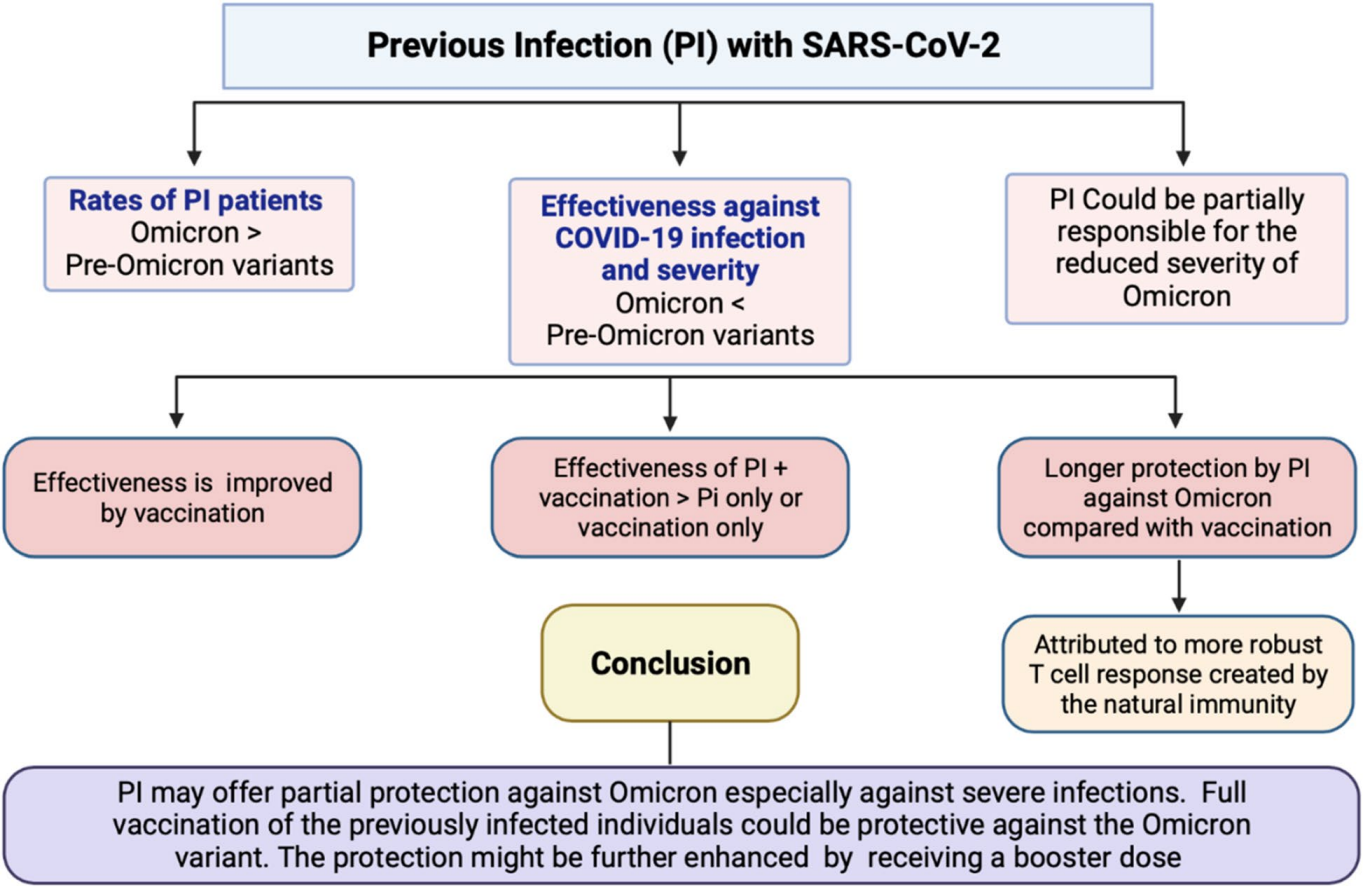
Summary of the effect of previous infection (PI) with any of the SARS-CoV-2 variants on the Omicron infections and its severe complications

Our review summarized recent studies that analyzed effectiveness of previous infection in protecting against the Omicron variant. Most of the studies reached a consensus that although previous infection provides some degree of immunity against Omicron reinfection, it is much lower in comparison to Delta. When evaluating vaccination status, being fully vaccinated with two doses was more protective against Delta than Omicron and receiving a booster dose served to provide additional protection against Omicron. Given recent emerging data on the newer variants, it is clear that neither vaccination nor previous infection alone provide optimal protection; hybrid immunity has demonstrated the best results in terms of protecting against either Omicron or Delta variants. However, additional research is needed to quantify how long immunity from vaccination versus previous infection lasts, and whether individuals will benefit from variant-specific vaccinations to enhance protection from infection (Fig. 3).

## Study limitations

This study has some limitations. Although several papers analyzed the effectiveness of previous infection in protecting against reinfection from the Omicron variant, some of them did not stratify the data based on vaccination status. Additionally, most studies did not stratify data based on time since vaccination and previous infection. This factor is important to consider as waning immunity overtime can provide a false impression of being protected against reinfection. This is especially relevant for Omicron, where antigenic modifications have been revealed to reduce antibody responsiveness after vaccination and previous infection. Since every country had its own timeline for COVID-19 waves, it can be argued that the data is not necessarily generalizable globally since there are other factors that influence rates of infection, such as public response to mask mandates and other personal protective factors. Additionally, certain countries had more prominent subvariants which may have significantly different characteristics to the main Omicron or Delta variant, and thus influence representativeness of this data in that population.

## Supplementary Information

**Additional file 1.**

**Additional file 2.**

## Data Availability

The data that supports the findings of this study are available in the supplementary material of this article.

## Abbreviations

WHO: World Health Organization
2019-nCoV: Novel coronavirus
SARS-CoV-2: Severe acute respiratory syndrome coronavirus 2
COVID-19: Coronavirus disease
VOC: Variant of Concern
CDC: Center for Disease Control and Prevention
nAb: Neutralizing antibody
mRNA: Messenger RNA
hACE2: Human angiotensin-converting enzyme 2
B.1.1.7: Alpha variant
SGTF: S-gene target failure
B.1.351: Beta variant
P.1: Gamma variant
B.1.617.2: Delta variant
B.1.1.529: Omicron variant
PRISMA: Preferred Reporting Items for Systematic reviews and MetaAnalysis
PIE: Previous Infection Effectiveness
HR: Hazard Ratio
NOS: Newcastle–Ottawa Quality Assessment Scale
PI: Previous Infection
CI: 95% Confidence interval
BA.1/BA.2: Omicron subvariants
ICU: Intensive Care Unit
CD8+: Cytotoxic T cells
RBD: Receptor Binding Domain
NTD: N-terminal protein
