# Supplementary material for: Role of previous infection with SARS-CoV-2 in protecting against omicron reinfections and severe complications of COVID-19 compared to pre-omicron variants: a systematic review
Source: BMC Infect Dis. 2023 Jun 26;23:432. doi: 10.1186/s12879-023-08328-3 (PMC10294418; doi:10.1186/s12879-023-08328-3)
Supplement: Supplementary file 1 — Additional file 1. [file 12879_2023_8328_MOESM1_ESM.docx]

Material for Appendix I:

**PubMed – Search run on March 6, 2022**

Omicron[tiab] OR "B.1.1.529"[tiab]

Filters Used: Publication Date – Custom Range: 2020/1/1 – Current.

**Medline (Ovid, 1946 – April 2021) - Search run on March 6, 2022**

1 | (Omicron OR "B.1.1.529").ti,ab.

2 | Limit 1 - 2020 to Current

**Embase (Ovid, 1974 – April 2021) - Search run on March 6, 2022**

1 | (Omicron OR "B.1.1.529").ti,ab.

2 | Limit 1 - 2020 to Current

**Scopus - Search run on March 6, 2022**

1 | TITLE-ABS(Omicron OR "B.1.1.529")

**Web of Science - Search run on March 6, 2022**

TS=(Omicron OR "B.1.1.529")

**Science Direct - Search run on March 6, 2022**

(Omicron OR "B.1.1.529")

Search results filtered to "Title, abstract or author-specified keywords"

**MedRxiv - Search run on March 6, 2022**

Title and Abstracts searched for: Omicron OR "B.1.1.529" (match all words) and posted between "01 Jan 2020 and Current"

**Lens.org - Search run on March 6, 2022**

(Omicron OR "B.1.1.529")

Filters Used: Date – 2020 to Current, Flags - Cited by Scholarly Works
