# Supplementary material for: Role of previous infection with SARS-CoV-2 in protecting against omicron reinfections and severe complications of COVID-19 compared to pre-omicron variants: a systematic review
Source: BMC Infect Dis. 2023 Jun 26;23:432. doi: 10.1186/s12879-023-08328-3 (PMC10294418; doi:10.1186/s12879-023-08328-3)
Supplement: Supplementary file 2 — Additional file 2. [file 12879_2023_8328_MOESM2_ESM.docx]

**Supplementary Table S1:** Rates of previously infected (PI) individuals in cohorts infected with different variants of SARS-CoV-2 and the effectiveness of previous infection in protecting against infection and or severe complications of COVID-19.

| **No.** | Study | **Country/Study type** | **Description** | **Negative cohorts**  **% (n/N)** | **Delta (or other) infected cohorts**  **% (n/N) or PIE** | **Omicron infected cohorts**  **% (n/N) or % (n) or PIE** | **P value** | **NOS Value** |
| --- | --- | --- | --- | --- | --- | --- | --- | --- |
| 1 | Eggink et al. [35] | Netherlands  Case only approach cohort study | Unvaccinated with PI | - | 1.4 %  (1295/93734) | 6.5% (5253/80615) | NR | 8 |
|  |  |  | Naïve (no PI no vaccine) | - | 37.1%  (34765/93734) | 27.4% (22071/80615) | NR |  |
|  |  |  | Primary vaccinated without PI | - | 61.5%  (57674/93734) | 66.1% (53291/80615) | NR |  |
| 2 | Shrestha et al. 90 [36] | USA  Retrospective cohort study | - | - | - | 2.9%  (88/4585) symptomatic  1.5%  (2/133) hospitalized | - | 7 |
| 3 | Hajjo et al. [47] | Jordan  Questionnaire based study | Reinfected within 90 days | - | - | 8.6 %  (43/500)  of which:  41.9% (18/43) asymptomatic  44.2% (19/43) mild  2.3% (1/43) moderate  2.3% (1/43) severe  9.3% (4/43) unspecified | - | 6 |
| 4 | Flacco et al. [55] | Italy  Retrospective cohort study | PI | - | 317 days of pre-Omicron period  (*n* = 116; 0.4 per day) | First 54 days of the Omicron wave  (*n* = 613; 11.4/day) | NR | 9 |
| 5 | Altarawneh et al. [56] | Qatar  Case control study | PIE overall | - | Alpha: 90.2% (60.2-97.6)  Beta: 85.7% (75.8-91.7)  Delta: 92.0% (87.9-94.7) | 56.0% (50.6-60.9) | NR | 9 |
|  |  |  | PIE adjusted for vaccine status | - | Alpha: 90.3% (60.4-97.6)  Beta: 85.1% (74.5-91.3)  Delta: 91.9% (87.8-94.7) | 55.9% (50.5-60.8) | NR |  |
|  |  |  | PIE vaccinated excluded | - | Alpha: 95.3% (66.0-99.3)  Beta: 85.4% (72.4-92.2)  Delta: 90.2% (81.9-94.6) | 61.9% (48.2-72.0) | NR |  |
|  |  |  | PIE against severe, critical, or fatal COVID-19 | - | Alpha: 69.4% (−143.6-96.2)  Beta: 88.0% (50.7-97.1)  Delta: 100% (43.3-100) | 87.8% (47.5-97.1) | NR |  |
| 6 | Smid et al. [57] | Czech Republic  Database based study | *PIE against infection  (overall) | - | Shortly after infection  95% (94–96)  After 6 months  83% (82–84) | Shortly after infection  68% (68–69)  After 6 months  13% (11–14) | NR | 6 |
|  |  |  | *PIE against infection  (not vaccinated) | - | 2–6 months after previous infection  93% (91–94)  7–10 months  91% (90%–92%),  11–14 months  86% (85–86)  ≥14 months  79% (77–81) | 2–6 months after previous infection  69% (68–69)  7–10 months  48% (46–50)  11–14 months  34% (33–35)  ≥14 months  17% (15–18) | NR |  |
|  |  |  | *PIE against hospitalization overall | - | <6 months  100% (no case)  >6 months  94% (91–96) | <6 months  73% (55–84)  >6 months  66% (54–75) | NR |  |
|  |  |  | *PIE against hospitalization  Full vaccination <2 month ago | - | <6 months  100% (no case)  >6 months  97% (91–99) | <6 months  100% (no case)  >6 months  94% (77–95) | NR |  |
|  |  |  | *PIE against hospitalization Full vaccination >2 month ago | - | <6 months  100% (no case)  >6 months  98% (98–100) | <6 months  93% (49–99)  >6 months  73% (78–99) | NR |  |
|  |  |  | *PIE against hospitalization  Booster <2 month ago | - | <6 months  100% (no case)  >6 months  99% (99–100) | <6 months  100% (no case)  >6 months  95% (78–99) | NR |  |
|  |  |  | *PIE against hospitalization  Booster > 2 month ago | - | <6 months  100% (no case)  >6 months  98% (85%–100%) | <6 months  71% (0–96)  >6 months  90% (64%–98%) | NR |  |
|  |  |  | *PIE against  oxygen therapy overall | - | <6 months  100% (no case)  >6 months  98% (95%–99%) | <6 months  81% (40%–94%)  >6 months  88% (72%–94%) | NR |  |
|  |  |  | *PIE against  ICU  overall | - | <6 months  100% (no case)  >6 months  97% (90%–99%) | <6 months  83% (0–98%)  >6 months  66% (15%–86%) | NR |  |
| 7 | Lewnard et al. [58] | USA  Cohort study | 15 December 2021 to 17 January 2022  (Documented PI) | - | 0.4 %  (84/23305) | 0.5%  (1,163/222688) | NR | 8 |
|  |  |  | 3 February to 17 March 2022  (Documented PI) | - | - | 0.6%  (75/12756) BA.1  0.4%  (7/1905) BA.2 | NR |  |
| 8 | Stegger et al. [59] | Denmark  Danish COVID-19 surveillance | Rate of second infection when previously infected with BA.1 | - | 0% (0/263) | 6.46% (17/263) BA.1  17.87% (47/263) BA.2 | NR | 8 |
|  |  |  | Rate of second infection when previously infected with BA.2 | - | 0% (0/263) | 0% (0/263) BA.1  1.14% (3/263) BA.2 | NR |  |
|  |  |  | Rate of second infection when PI with Delta | - | 11.4% (30/263) | 9.88% (26/263) BA.1  53.23% (140/263) BA.2 | NR |  |
|  |  |  | Rate of second infection when PI (overall) | - | 11.4% (30/263) | 12.54% (33/263) BA.1  72.24% (190/263) BA.2 | NR |  |
| 9 | Andrews et al. [60] | UK  Test-negative case–control | Rate of PI out of the Delta and Omicron | 16.5% (260,073/1572621) | 1.8% (3,754/204154) | 11.1% (98,150/886774) | NR | 8 |
| 10 | Andeweg et al. [61] | Netherlands  Cohort study  Of the infected people the presented % were: | Naïve  (no PI no vaccine) | 30.2% (90,945/300849) | 52.8% (21,042/39889) | 24.7% (3440/13915) BA.1 | NR | 9 |
|  |  |  | PI unvaccinated | 4.2% (12,691/300849) | 1.6% (638/39889) | 5.3% (739/13915) BA.1 | NR |  |
|  |  |  | First start primary vaccination, then infection | 1.1% (3406/300849) | 0.2% (76/39889) | 1.7% (240/13915) BA.1 | NR |  |
|  |  |  | First infection, then primary vaccination | 2.3% (7002/300849) | 0.3% (139/39889) | 2.1% (293/13915) BA.1 | NR |  |
|  |  |  | PI, booster | 0.3% (918/300849) | 0% (2/39889) | 0.5% (65/13915) BA.1 | NR |  |
| 11 | Smith-Jeffcoat et al. [37] | USA  Cohort (convention attendees) | PI | 0% (0/7) | - | 6.25% (1/16) | NR | 7 |
| 12 | Espenhain et al. [38] | Denmark  Data from the routine Danish surveillance of COVID-19 | PI | - | 0.9% (160/19137) | 4.3% (34/785) | NR | 9 |
| 13 | Nunes et al. [39] | South Africa  Cohort study | PI | 41.6% (101) | - | 27.9% (53) | 0.003 | 9 |
|  |  |  | Naïve (no PI no vaccine) | 10.8% (23) | - | 13.2% (23) | NR |  |
|  |  |  | PI, no vaccines | 6.5% (14) | - | 5.2% (9) | NR |  |
|  |  |  | No PI, J&J | 48.6% (104) | - | 56.9% (99) | NR |  |
|  |  |  | PI + J&J | 34.1% (73) | - | 24.7% (43) | NR |  |
| 14 | Sharma et al. [40] | Rajasthan, India  Cohort study | PI | - | - | 43.2% (126) | - | 9 |
| 15 | Spensley et al. [41] | UK  Cohort study | PI overall | 53.5% (516/965) | - | 43.4% (63/145) | 0.024 | 9 |
|  |  |  | PI + ChAdOx1 | - | - | 46.2% (226/489) | 0.75 |  |
|  |  |  | PI + BNT1262b2 | - | - | 47.2% (260/551) | 0.75 |  |
| 16 | Davies et al. [42] | South Africa  Cohort study | HR for PI (vs. None) | - | - | Death  1.10 (0.63 - 1.92)  Severe hospitalization or death  0.60 (0.37 - 0.98)  Hospitalization or death  0.28 (0.19 - 0.40) | - | 9 |
|  |  |  | PI | - | Wave 3 (Delta, 20/5/2021 to 23/6/2021)  3.2% (140/4403) | Wave 4 (Omicron, 14/11/2021 to 11/12/2021)  11.3% (580/5144) | - |  |
| 17 | MMWR [43] | USA  Cohort study | PI | - | - | 14% (6/43) | - | 6 |
| 18 | Madhi et al. [44] | South Africa  seroepidemiologic survey | PI | - | - | 2.8% (195) | - | 9 |
| 19 | Kahn et al. [45] | Sweden  Cohort study | Unvaccinated (0-1 dose, n=23217) | - | 2.7% (265/9680) | 7.1% (562/7861) PI | NR | 9 |
|  |  |  | Vaccinated (2-3 dose, n=32052) | - | 2.3% (94/4031) | 6.3% (1376/21678) PI | NR |  |
| 20 | Kislaya et al. [46] | Portugal  Data linkage  Case-case study | Naïve  (no PI no vaccine) | - | 10.6% (888) | 6% (315) | NR | 9 |
|  |  |  | PI unvaccinated | - | 1.3% (108) | 6.2% (327) | NR |  |
|  |  |  | No PI partial vaccination | - | 1.3% (112) | 1.3% (68) | NR |  |
|  |  |  | PI partial/complete vaccination | - | 0.3% (25) | 0.84% (44) | NR |  |
|  |  |  | No PI complete vaccination | - | 88.6% (7245) | 85.7% (4515) | NR |  |
| 21 | Maisa et al. [48] | France  Questionnaire based | PI | - | - | 14% (39/278)  2% hospitalized (7/294) – of these 7, 2 PI | - | 8 |
| 22 | Peralta-Santos et al. [49] | Portugal  Cohort Study  National network group of laboratories | PI | - | 1.6% (146/9397) | 6.8% (449/6581) | <0.001 | 9 |
| 23 | Qassim et al. [50] | Qatar  Cross sectional study | No PI | - | - | 90.8% (141,839) | - | 9 |
|  |  |  | PI <90 days before the study | - | - | 0.4% (560) | - |  |
|  |  |  | PI overall | - | - | 8.8% (13,803) | - |  |
| 24 | Wolter et al. [51] | South Africa  Data linkage study | **PI | - | 4.5% (43/948) (non SGTF) | 10.4% (1100/10 547)  (SGTF) | 0.18 | 9 |
| 25 | Ward et al. [52] | UK  Data linkage  **PI |  | - | 1% (2211/221146) | 6.6% (53724/814003) | NR | 9 |
| 26 | Garg et al. [53] | India  Cohort study |  | - | 8.2% (6/182) | 17.1% (14/82) | NR | 4 |
| 27 | Krutikov et al. [54] | UK  Prospective cohort study |  | - | Pre-Omicron  4.3% (17/400) | 12.7% (236/1864) | <0.0001 | 8 |

N: Total number of subjects in the cohort

n: number of subjects who were PI or as specified in each row.

§ All values are previous infection effectiveness (PIE) with 95% confidence intervals stratified by the vaccination status.

# Effectiveness of PI (95% confidence interval) against hospitalization, oxygen therapy, or intensive care stratified by the vaccination status a time of vaccination

*A positive test during the first 2 months after an infection is not considered a reinfection by definition and.

**Reinfection was defined by an individual having at least one positive SARS-CoV-2 test more than 90 days before the current positive test.

SGTF: samples with S gene target failure (strongly indicative of an Omicron variant).
